# Associated factors and sex differences in condom non-use among adolescents: Brazilian National School Health Survey (PeNSE)

**DOI:** 10.1186/s12978-020-00987-8

**Published:** 2020-09-07

**Authors:** Matias Noll, Priscilla Rayanne E Silva Noll, Jéssica Menezes Gomes, José Maria Soares Júnior, Erika Aparecida Silveira, Isabel Cristina Esposito Sorpreso

**Affiliations:** 1 Instituto Federal Goiano (IF Goiano), Goiás, Brazil; 2 Departamento de Clínica Médica, Faculdade de Medicina, Universidade Federal de Goiás (UFG), Goiás, Brazil; 3 Disciplina de Ginecologia, Departamento de Obstetrícia e Ginecologia, Faculdade de Medicina FMUSP, Universidade de São Paulo, São Paulo, SP Brazil

**Keywords:** Students, Condom use, Sexual risk behavior, Adolescent health

## Abstract

**Background:**

Condom non-use among sexually active adolescents is a major cause of unintended pregnancy and sexually transmitted infections. In order to promote condom use, it is essential to understand factors associated with condom non-use.

**Aim:**

Our aim was to evaluate sex differences and associated factors of condom non-use based on the nationally representative Brazilian National School Health Survey.

**Methods:**

The study participants were 100,962 adolescents 13–18 years old, 9th graders from both public and private schools throughout Brazil. The following factors were considered as explanatory group variables for the outcome of condom non-use among adolescents: school and health service, sexual behavior, substance use, and self-reported body and health perception. Poisson regression model was performed.

**Results:**

Of the total students, 28% (*n* = 28,157) had had sexual intercourse at least once. (boys, 37.1%; girls, 19.5%). Of these, 69.2% had used condoms the last time they had intercourse (girls: 68%; boys: 69.9%). The variables associated with condom non-use for both sexes were not having accessed a health service or approached a health professional for health-related care; not having received pregnancy prevention counseling or guidance on AIDS or STI prevention at school; early sexual initiation; no additional contraception method; substance use; feeling alone; not being satisfied with their own body; feeling fat or thin; and poor self-reported health. The number of sexual partners was also associated with condom non-use; however, contrasting behavior was indicated between sexes. A higher number of sexual partners indicated less use of condoms among girls, while for boys, a higher number of sexual partners indicated higher condom use.

**Conclusion:**

High condom non-use appears to be associated with lack of health care access and sexual health education, poor sexual practices, substance use, and poor self-perception, indicating areas for health promotion programs.

## Plain English summary

Condom non-use among sexually active adolescents is associated with unintended pregnancy (UP) and sexually transmitted infections (STIs). Studies on this topic may contribute to education programs about the importance of condom use for UP and STI prevention and are necessary for adequate contraceptive use and preventive counseling among adolescents.

This study evaluated some factors and sex differences in condom non-use among adolescents. Of the 100,962 students who voluntarily agreed to participate and provided written informed consent, 48,790 (48.3%) were boys and 52,172 (51.7%) were girls. From this total number, 28% had had sexual intercourse previously. Of these, 69.2% had used condoms during their last sexual encounter.

Our results also showed that lack of health care access and sexual health education, poor sexual practices, substance use (smoking, alcohol intake, and drug use), and poor self-perception were related to high condom non-use among adolescents in Brazil.

## Introduction

Condom non-use among sexually active adolescents is associated with unintended pregnancy (UP) and sexually transmitted infections (STIs) [1]. Adolescent pregnancy should be avoided because it is associated with poor maternal and child health outcomes and linked to poor socioeconomic status and educational worst consequences [2–5]. Although several contraceptive methods prevent pregnancy, to prevent STIs such as gonorrhea, non-gonococcal urethritis, trichomoniasis, genital herpes, and HIV, only barrier methods are effective, namely, condom use [6].

Preventive behaviors exhibited in early adolescence are strong determinants of later healthy behaviors [7]. Shafii et al. [7] and Brahmbhatt et al. [8] verified that adolescents who use a condom during their sexual debut are more likely to use condoms during their most recent intercourse and have decreased risk of pregnancy. Therefore, understanding the associated factors of condom non-use is essential to improve health and educational programs [9].

Most studies worldwide to date that have evaluated the associated factors of condom non-use of adolescents have focused on demographics [10, 11]; substance use (alcohol, tobacco, and prohibited substances) [10, 12]; and sexual activity factors (number of sexual partners, age of sexual debut) [7, 13]. Most of these studies, however, have also used local or non-representative samples [14, 15]. Given this background, we chose to use a nationally representative study of Brazil in order to expand the range of associated factors related to condom non-use and to analyze related socio-environmental factors (health education and access to health services) and self-reported health, neither of which had yet been investigated. Moreover, as recently suggested by Harper et al. [13], we analyzed differences between boys and girls.

A recent nationally representative study [16] on Irish adolescents focused on young people as a distinct population subgroup with unique influences on their sexual health, requiring targeted interventions and policy. From Latin American countries, however, to the best of our knowledge, there have been no nationally representative studies evaluating a wide range of factors associated with condom non-use. Therefore, the aim of the present study was to evaluate associated factors and gender differences in condom non-use based on the Brazilian National School Health Survey. Education programs on the importance of condom use for STI and UP prevention are critical and necessary for adequate contraceptive use and preventive counseling among adolescents [2], and the findings of the present study are expected to help improve behavior-focused programs at school.

## Methods

This study used the Brazilian National School Health Survey (PeNSE) database [17]. This survey of students from public and private schools across Brazil was conducted through a partnership between the Ministry of Health and the Brazilian Institute of Geography and Statistics (Instituto Brasileiro de Geografia e Estatística; IBGE). The PeNSE was approved by the National Commission on Ethics in Research (Comissão Nacional de Ética em Pesquisa; CONEP) of the National Health Council, which regulates and approves health research involving human participants (CONEP resolution no. 1,006,467; March 30, 2015) [18]. PeNSE data collection assesses several health outcomes, and many studies are dedicated to analyzing aspects of those outcomes [19–24].

The survey we used was conducted in 2015 and evaluated enrolled students and regular 9th graders attending Brazilian public and private schools. This sample of adolescents adequately represents youth across Brazil, including all 27 federative units (26 states with capitals and municipalities as well as the Federal District; IBGE). The study data were made available by the IBGE in 2016 [18].

The sample was sized in order to estimate the parameters for each of the 26 capitals and the Federal District formed by the five regions of the country (North, Northeast, Southeast, South, and Midwest). Samples of the geographic levels comprising capitals and municipalities were random and equiprobabilistic. The following parameters were used for sample calculation: 0.03% maximum error, 95% confidence level, and prevalence of 0.5. Further details on the sampling process and the topics investigated can be found in the PeNSE publication [18, 19].

**Fig. 1:**
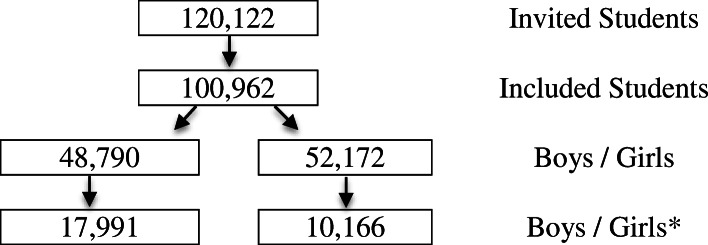
Adolescent sample flow chart from the Brazilian National School Health Survey (PeNSE). * Students who had sexual intercourse previously. Sample evaluated in this study

Overall, 120,122 students who were enrolled in and attended one of 4159 classes across 3040 schools were included in the 2015 sample. Of these, 100,962 students completed the survey on the sampling day. As all the students in the sampled classes were invited to respond to the survey questionnaire, there was a sample loss of approximately 16%. This study included the data of adolescents between 13 and 18 old of both genders, who were classified as 9th graders in either public or private schools throughout Brazil from April to September 2015 (Fig. 1).

All the students who agreed to participate voluntarily provided written informed consent. Students were told that they could leave the study at any time if they chose not to participate in any of the procedures [25]. This study was conducted in accordance with the Strengthening the Reporting of Observational Studies in Epidemiology (STROBE) checklist [26].

Data collection, involving a validated self-administered survey [18], was performed using smartphones distributed by the IBGE technician to the students who were in class on the day of the interview. The analytic sample was restricted to currently sexually active students, assessed with the question: “Have you ever had sexual intercourse?”. The response options were *Yes* and *No*.

In this study, we considered condom use during the most recent occasion of sexual intercourse as the outcome variable, assessed using the question: “The last time you had sexual intercourse, did you or your partner use a condom?” Response options were *Yes* and *No.*

The explanatory variables groups were as follows:

- **Socioeconomic** (Municipality, School, Age, and Mother’s Level of Education);
- **School and health service** (In the last 12 months, have you access to a health service or approached a health professional for health-related care?; At school, have you received guidance on pregnancy prevention?; At school, have you received counseling about AIDS or other STIs?);
- **Sexual behavior** (Age of first sexual intercourse; Number of sexual partners; Did you or your partner use an additional contraception method the last time you had sexual intercourse?);
- **Substance use** (Have you ever smoked in your life?; During the last 30 days, how many days did you smoke?; Have you ever drunk alcohol in your life?; During the last 30 days, how many days did you drink?; In your life, how many times did you drink until you got drunk?; Have you ever used drugs in your life (marijuana, cocaine, crack)?; During the last 30 days, how many days did you use drugs?)
- **Self-reported general health** (Feeling alone; Self-reported health; Feelings about the body; Body weight perception).

Data were analyzed using descriptive statistics and the Wald chi-square test of association (bivariate analysis) for the outcome of condom non-use. We performed each analysis separately for each sex, specifically in relation to sexual behavior, because adjusting the model by sex might not have provided reliable findings.

Four variable groups were considered in a Multiple Poisson regression model analysis with robust variance [27]: School and health service, Sexual behavior, Substance use, and Self-reported general health. The assumptions required to perform the Poisson regression were respected [27]. Explanatory variables were adjusted by confounding variables (Municipality, School, Age, Mother’s Level of Education, and Additional contraception method use). Methodological and statistical studies support the inclusion of variables with theoretical grounds in statistical analyses [27–29]. The effect measure was the prevalence ratio with its respective 95% confidence intervals (α = 0.05). Statistical analyses were performed using the Statistical Package for Social Sciences (SPSS 20.0, IBM, Armonk, NY, US).

## Results

The sample for this research included 100,962 students: 48,790 (48.3%) boys and 52,172 (51.7%) girls. From this total number, 37.1% of boys and 19.5% of girls had had sexual intercourse previously, and only the data for these students (*n* = 28,157; 28%) were included in the present study (Fig. 1). Table 1 presents the sample description data for the evaluated variables.

**Table 1:** Socioeconomic characteristics, health and school services, sexual and risk behavior, and self-reported general health among sexually initiated adolescents interviewed by PENSE, Brazil (*n* = 28,157)

Variables	Total %	Male (***n*** = 17,991) %	Female (***n*** = 10,166) %
**Socioeconomic**			
Municipality			
Not capital	51.1	51.3	50.8
Capital	48.9	48.7	49.2
School			
Public	88.3	86.9	90.8
Private	11.7	13.1	9.2
Age			
13 years	6.7	7	6.2
14 years	38.4	38.1	38.7
15 years	30.6	30.1	31.4
16 years	15.8	16.2	15.1
17 years	6.7	6.8	6.4
18 years	1.9	1.8	2.2
Mother’s Level of Education			
No school	9.2	8.4	10.7
Primary Education	35.5	33.1	39.5
High School	31.2	31.5	30.7
Undergraduate coursework	24.1	27	19
**School and health service**			
In the last 12 months, have you had access to a health service or approached a health professional for health-related care?			
Yes	55.5	52.7	60.5
No	44.5	47.3	39.5
At school, have you received pregnancy prevention counseling?			
Yes	80.3	81.2	78.8
No	19.7	18.8	21.2
At school, have you received counseling about AIDS or other STIs?			
Yes	88.2	88.2	88.1
No	11.8	11.8	11.9
**Sexual behavior**			
Age of first sexual intercourse			
≤ 12 years	26.2	33.8	12.6
13 to 14 years	55.1	50.6	62.9
≥ 15 years	18.7	15.4	24.5
Number of sexual partners			
1	36	26.5	52.9
2 to 3	32.3	34	29.4
4 or more	31.6	39.5	17.7
Additional contraception method use			
No	57.3	59.8	53.5
Yes, hormonal contraception method^a^	29.6	25.3	36.2
Yes, non-hormonal contraception method^b^	13.1	15	10.3
**Risk behaviors**			
Smoked at least once			
No	60.5	62.9	56.4
Yes	39.5	37.1	43.6
During the last 30 days, how many days did you smoke?^c^			
None	65.5	66.1	64.5
1 to 2 days	16.9	15.8	18.4
3 to 9 days	8.6	8.4	8.8
10 or more days	9.1	9.6	8.3
Drank alcohol at least once			
No	22.7	26	17
Yes	77.3	74	83
During the last 30 days, how many days did you drink?^c^			
None	47.3	49.3	44.2
1 to 2 days	27.5	26.4	29.2
3 to 9 days	16.5	15.9	17.6
10 or more days	8.7	8.5	9
In your life, how many times did you drink until you got drunk?^c^			
None	46.5	48.5	43.2
1 to 2 days	31.3	29.1	34.8
3 to 9 days	14.5	14.1	15.1
10 or more days	7.7	8.3	6.8
Used drugs at least once (marijuana, cocaine, crack)			
No	78.3	79.8	75.8
Yes	21.7	20.2	24.2
During the last 30 days, how many days did you use drugs?^c^			
None	50.6	49.8	51.9
1 to 2 days	23.2	22.3	24.6
3 to 9 days	14.5	14.8	14
10 or more days	11.6	13.1	9.5
**Self-reported general health**			
Feel alone			
Never	34.2	43.1	18.6
Rarely	20.1	21.4	17.9
Sometimes	27.6	24.2	33.7
Most of the time or always	18	11.4	29.8
Body weight perception			
Very thin	5.5	5.1	6.3
Thin	19.5	21	16.9
Normal	58.7	61.3	54
Fat	14.1	11.1	19.3
Very fat	2.2	1.5	3.5
Feelings about the body			
Very satisfied	34.2	37.7	28.1
Satisfied	40	41.2	38
Indifferent	9.5	9.4	9.8
Unsatisfied	11.9	8.4	18.1
Very unsatisfied	4.3	3.3	6.1
Self-reported state of health			
Very good	38.6	44.6	28.1
Good	29.4	29.1	30
Regular	21.5	17	29.5
Poor	5.2	4	7.2
Very poor	5.3	5.3	5.2

*PeNSE* Pesquisa Nacional de Saúde do Escolar (National School Health Survey); *AIDS* Acquired Immunodeficiency Syndrome; *STI* Sexually Transmitted Infections

^a^ Hormonal contraception method: contraceptive pill, injection or patch, hormonal intrauterine device

^b^Non-hormonal contraception method: intrauterine device, diaphragm, or other

^c^ Only asked of those to whom it was relevant

Regarding condom use during the most recent sexual intercourse, 69.2% of the participants indicated that they had used condoms (68% for females and 69.9% for males). The results of the prevalence of non-use condom for socioeconomic variables are presented in Table 2. Results from school and health services, sexual behavior, and self-reported general health variables are presented in Table 3.

**Table 2:** Prevalence of socioeconomic variables and association with condom non-use in Brazilian national adolescent sample

Variables	Condom non-use					
	Male			Female		
	%	RP (CI 95%)	***p***	%	RP (CI 95%)	***p***
**Socioeconomic**						
Municipality						
Not capital	28.4	1	< 0.001	30.2	1	< 0.001
Capital	31.9	**1.13(1.08–1.18)**		33.8	**1.12(1.6–1.18)**	
School						
Public	30.2	1	0.533	31.9	1	0.645
Private	29.5	0.98(0.91–1.05)		32.7	1.02(0.93–1.13)	
Age						
13–14 years	32.4	1	< 0.001	30.5	1	0.004
15–16 years	28.3	**0.88(0.84–0.91)**		32.7	**1.07(1.01–1.14)**	
17–18 years	27.6	**0.85(0.78–0.93)**		35.7	**1.17(1.06–1.29)**	
Mother’s Level of Education						
No school	27.2	1	0.134	32.1	1	0.296
Primary Education	30.6	1.13(1.00–1.25)		33	1.03(0.92–1.15)	
High School	29.3	1.08(0.97–1.20)		32.9	1.02(0.91–1.15)	
Undergraduate coursework	29.2	1.07(0.96–1.20)		30.3	0.95(0.83–1.07)	

*PeNSE* Pesquisa Nacional de Saúde do Escolar (National School Health Survey); *PR* prevalence ratio; *CI* confidence interval

**Table 3:** Prevalence of school and healthcare, sexual behavior, risk behaviors, and self-reported health variables and their association with condom non-use in Brazilian national adolescent sample

Variables	Condom non-use				Adjusted^**d**^ analysis of condom non-use			
	Male		Female		Male		Female	
	%	RP (CI 95%)	%	RP (CI 95%)	Adj RP (CI 95%)	***p***	Adj RP (CI 95%)	***p***
**School and health service**								
In the last 12 months, have you had access to a health service or approached a health professional for health-related care?								
Yes	26.7	1	30.2	1	1	< 0.001	1	< 0.001
No	34.0	**1.28(1.22–1.33)**	34.8	**1.15(1.09–1.22)**	**1.22(1.17–1.27)**		**1.12(1.06–1.19)**	
At school, have you received pregnancy prevention counseling?								
Yes	29.4	1	30.7	1	1	< 0.001	1	< 0.001
No	33.7	**1.15(1.08–1.21)**	36.8	**1.20(1.12–1.28)**	**1.14(1.08–1.20)**		**1.19(1.12–1.27)**	
At school, have you received counseling about AIDS or other STIs?								
Yes	29.3	1	31.3	1	1	< 0.001	1	< 0.001
No	34.8	**1.19(1.11–1.27)**	37	**1.18(1.09–1.28)**	**1.17(1.09–1.25)**		**1.17(1.08–1.27)**	
**Sexual behavior**								
Age of first sexual intercourse								
≥ 15 years	22.2	1	26.2	1	1	< 0.001	1	< 0.001
13 and 14 years	24.1	1.09(1.00–1.18)	31.8	**1.21(1.13–1.31)**	1.05(0.96–1.14)		**1.39(1.28–1.51)**	
≤ 12 years	43.1	**1.94(1.80–2.10)**	44.7	**1.70(1.56–1.87)**	**1.78(1.64–1.93)**		**1.92(1.74–2.11)**	
Number of sexual partners								
1	34.6	1	27.3	1	1	< 0.001	1	< 0.001
2 to 3	30.2	**0.87(0.83–0.93)**	33.5	**1.23(1.15–1.31)**	**0.88(0.83–0.93)**		**1.21(1.13–1.29)**	
4 or more	27.1	**0.79(0.74–0.83)**	43.5	**1.59(1.49–1.71)**	**0.84(0.80–0.89)**		**1.55(1.45–1.66)**	
Additional contraception method use								
No	40.1	1	39.8	1	**1**	< 0.001	1	< 0.001
Yes, hormonal contraception method^a^	17.3	**0.43(0.4–0.47)**	25.4	**0.64(0.60–0.68)**	**0.44(0.41–0.47)**		**0.63(0.59–0.68)**	
Yes, non-hormonal contraception method^b^	16.7	**0.42(0.38–0.46)**	22	**0.55(0.49–0.62)**	**0.42(0.38–0.46)**		**0.55(0.49–0.63)**	
**Risk behaviors**								
Smoked at least once								
No	26.1	1	25.8	1	1	< 0.001	1	< 0.001
Yes	36.7	**1.41(1.34–1.47)**	40	**1.55(1.46–1.64)**	**1.39(1.33–1.45)**		**1.49(1.41–1.58)**	
During the last 30 days, how many days did you smoke?^c^								
None	36.8	1	40.1	1	1	0.047	1	< 0.001
1 to 2 days	34.2	0.93(0.85–1.02)	36.7	0.92(0.83–1.01)	0.96(0.88–1.05)		0.92(0.83–1.01)	
3 to 9 days	39.6	1.07(0.96–1.20)	37	0.92(0.80–1.06)	1.10(0.98–1.23)		0.93(0.82–1.07)	
10 or more days	38.3	1.04(0.93–1.16)	48.9	**1.21(1.09–1.37)**	**1.11(1.02–1.24)**		**1.24(1.11–1.38)**	
Drank alcohol at least once								
No	24.7	1	22	1	1	< 0.001	1	< 0.001
Yes	32	**1.30(1.22–1.37)**	34	**1.55(1.41–1.70)**	**1.28(1.21–1.36)**		**1.51(1.37–1.65)**	
During the last 30 days, how many days did you drink?^c^								
None	32.9	1	32.2	1	1	0.001	1	< 0.001
1 to 2 days	29.3	**0.89(0.84–0.95)**	32.6	1.01(0.94–1.09)	**0.92(0.86–0.98)**		1.03(0.96–1.10)	
3 to 9 days	31.4	0.95(0.89–1.03)	36.9	**1.15(1.06–1.24)**	1.00(0.93–1.07)		**1.16(1.08–1.26)**	
10 or more days	34.8	1.06(0.97–1.16)	40.7	**1.26(1.15–1.39)**	**1.14(1.05–1.25)**		**1.30(1.18–1.42)**	
In your life, how many times did you drink until you got drunk?^c^								
None	30.2	1	28	1	1	< 0.001	1	< 0.001
1 to 2 days	32.9	**1.09(1.03–1.15)**	36.2	**1.29(1.20–1.38)**	**1.10(1.04–1.17)**		**1.28(1.19–1.37)**	
3 to 9 days	33.4	**1.11(1.03–1.19)**	40.1	**1.43(1.31–1.56)**	**1.15(1.07–1.24)**		**1.43(1.31–1.55)**	
10 or more days	36.0	**1.19(1.10–1.30)**	45.6	**1.63(1.47–1.80)**	**1.28(1.17–1.39)**		**1.63(1.48–1.81)**	
Used drugs at least once (marijuana, cocaine, crack)								
No	28.7	1	28.7	1	1	< 0.001	1	< 0.001
Yes	35.6	**1.24(1.18–1.31)**	42.3	**1.47(1.39–1.56)**	**1.25(1.19–1.32)**		**1.45(1.37–1.54)**	
During the last 30 days, how many days did you use drugs?^c^								
None	35.7	1	41.9	1	1	0.056	1	0.077
1 to 2 days	32.6	0.91(0.81–1.03)	40.8	0.97(0.87–1.09)	0.92(0.82–1.04)		0.97(0.86–1.08)	
3 to 9 days	35.7	1.00(0.87–1.14)	41.4	0.99(0.86–1.14)	1.02(0.89–1.17)		1.01(0.88–1.16)	
10 or more days	40.3	1.13(0.99–1.28)	49.8	**1.19(1.03–1.37)**	1.13(0.99–1.28)		1.18(0.99–1.35)	
**Self-reported general health**								
Feel alone								
Never	26.6	**1**	27.8	**1**	**1**	< 0.001	**1**	< 0.001
Rarely	29.2	**1.10(1.03–1.17)**	32	**1.15(1.04–1.28)**	**1.06(0.99–1.13)**		**1.12(1.02–1.24)**	
Sometimes	32.3	**1.21(1.14–1.29)**	31.8	**1.15(1.05–1.25)**	**1.18(1.12–1.25)**		**1.12(1.02–1.21)**	
Most of the time or always	40.1	**1.51(1.41–1.61)**	34.9	**1.26(1.15–1.37)**	**1.43(1.33–1.52)**		**1.21(1.11–1.33)**	
Body weight perception								
Very thin	35.4	**1.26(1.14–1.39)**	33.9	1.11(0.98–1.25)	**1.26(1.15–1.37)**	< 0.001	1.11(0.99–1.25)	0.017
Thin	32.8	**1.17(1.10–1.24)**	33.9	**1.11(1.03–1.20)**	**1.14(1.08–1.20)**		**1.09(1.01–1.17)**	
Normal	28.1	1	30.4	1	1		1	
Fat	32.2	**1.15(1.07–1.23)**	33.5	**1.10(1.02–1.18)**	**1.12(1.04–1.20)**		**1.08(1.01–1.16)**	
Very fat	45	**1.60(1.38–1.85)**	36.7	**1.20(1.04–1.39)**	**1.58(1.38–1.82)**		**1.20(1.0–1.38)**	
Feelings about the body								
Very satisfied	26.3	1	27.6	1	1	< 0.001	1	< 0.001
Satisfied	30.1	**1.15(1.09–1.21)**	31.8	**1.15(1.09–1.24)**	**1. 09(1.04–1.15)**		**1.14(1.05–1.22)**	
Indifferent	36.5	**1.39(1.29–1.50)**	34.2	**1.24(1.12–1.38)**	**1.33(1.23–1.44)**		**1.21(1.09–1.35)**	
Unsatisfied or very unsatisfied	37.8	**1.44(1.34–1.55)**	36.6	**1.33(1.22–1.44)**	**1.36(1.26–1.45)**		**1.29(1.19–1.39)**	
Self-reported state of health								
Very good	26.1	1	25.5	1	1	< 0.001	1	< 0.001
Good	31.3	**1.20(1.14–1.27)**	33.8	**1.32(1.22–1.44)**	**1.15(1.09–1.22)**		**1.28(1.18–1.39)**	
Regular	36.7	**1.41(1.32–1.50)**	34.6	**1.35(1.25–1.47)**	**1.36(1.28–1.44)**		**1.31(1.21–1.41)**	
Poor or very poor	34.3	**1.32(1.22–1.42)**	36.5	**1.43(1.30–1.57)**	**1.31(1.21–1.41)**		**1.39(1.26–1.53)**	

*PeNSE* Pesquisa Nacional de Saúde do Escolar (National School Health Survey); *AIDS* Acquired Immunodeficiency Syndrome; *STI* Sexually Transmitted Infections

Explanatory variables were adjusted by confounding variables (socioeconomic variables) in a Poisson regression model-based analysis with robust variance. The effect measure was the prevalence ratio (PR) with its respective 95% confidence intervals (CIs) (α = 0.05). Bolded *p*-values denote statistical significance (*p* < 0.05).

^a^Hormonal contraception method: contraceptive pill, injection or patch, hormonal intrauterine device

^b^Non-hormonal contraception method: intrauterine device, diaphragm, or other

^c^Only asked of those to whom it was relevant

^d^Variables adjusted by Municipality, School, Age, Mother’s Level of Education, and Other contraceptive method (except condom)

After inserting the variables in the adjusted analysis, the following variables were associated with condom non-use for both sexes: had not accessed a health service or approached a health professional for health-related care; had not received pregnancy prevention counseling or guidance on AIDS or STI prevention at school; early sexual initiation; no additional contraception method (hormonal or non-hormonal); substance use (smoking, alcohol intake, and drug use); feeling alone; not being satisfied with their own body; feeling fat or thin; and poor self-reported health (Table 3). The number of sexual partners was also associated with condom non-use; however, we verified contrasting behavior between genders. A higher number of sexual partners indicated less condom use for girls, whereas for boys, a higher number of sexual partners indicated higher condom use.

## Discussion

Our study evaluated sex differences and several factors associated with condom non-use from a large and representative country sample, and it was the first study to do so in the Latin American context [14]. Our main results show that for both sexes, the factors associated with condom non-use are (a) not accessing a health service or approaching a health professional for health-related care and not having received pregnancy prevention counseling or guidance on AIDS or STI prevention at school, (b) early sexual initiation and substance use, and (c) poor self-reported health. In short, high condom non-use in Brazilian adolescents appears to be associated with lack of health care access and sexual health education, poor health perception, and risky behaviors such as unhealthy or poor sexual behavior and substance use, indicating areas for health promotion programs.

The prevalence of sexual intercourse indicated that 37.1% of boys and 19.5% of girls were sexually initiated. Similar results are offered by the current literature. Young, Burke, and Gabhainn [16] conducted a nationally representative study in Ireland using a self-completed questionnaire for 4494 schoolchildren aged 15–18 years and found that 25.7% of boys and 21.2% of girls were sexually initiated. Australian government high schools reported that 34.4% of the students had engaged in sexual intercourse at least once [30]. Harper et al. [13] evaluated US high school students and found that the percentage of currently sexually active students ranged around 35.0%. Our results are similar to the existing literature, despite the differences between boys and girls.

Regarding condom use during the most recent intercourse, our results indicated similar results between sexes: 67.9% of female students and 69.9% of male students had used condoms. Although similar results were found between genders for schoolchildren in Ireland aged 15–18 years [16], a much higher condom use (80%) at last intercourse was reported. In a contrasting finding, results from a study on US adolescents [31] showed lower condom use at last sexual intercourse (≅55%). Moreover, another study [13] on US high school students offered concerning results, indicating that condom use during last sexual intercourse declined significantly in 2005 compared to 2003 in both female students (57 to 52%) and male students (69 to 62%).

Our results indicated that substance use (smoking, drinking, and taking drugs) was strongly related to condom non-use. Shrier et al. [31] found that boys and girls who had zero alcohol consumption before the last intercourse demonstrated higher condom use. Thamotharan et al. [32] found that not using condoms in the last relationship was associated with high consumption of alcohol in the last 30 days. The findings of Green et al. [12] suggest that specific patterns of alcohol and marijuana use during adolescence are associated with a higher risk of sexually risky behaviors and adverse sexual outcomes in young adulthood, including having sex without a condom. Hansen et al. [33] verified that early smoking initiation was related to less condom use. Thamotharan et al. [32] found that more days of smoking per month, more cigarettes per day, and daily smoking were associated with not using condoms in the last relationship. Casola et al. [9] and Thamotharan et al. [32] found that marijuana use among adolescents was a statistically significant risk factor for contraception non-use.

Smoking, consuming alcohol until getting drunk, and using drugs may make the adolescent lose track of rationality, and those practices as well as the coexistence of sexual behavior influences and norms regarding sexual behavior (e.g., condom use-related stigma) may be contributing to declining use of condoms. A recent meta-analysis assessing the relationship between marijuana and condom use at instances of sexual intercourse [15] found a statistically significant relationship between marijuana use and lower condom use among adolescents. Cocaine use may create ideal conditions for risk behavior by acutely increasing behavioral processes, perhaps interactive behavior, including sexual desire and sexual delay discounting (detrimental effect of delay on condom use) [34]. These findings are not entirely surprising given the existing evidence indicating that adolescent risk behaviors tend to co-occur simultaneously because of shared social determinants and risk [35].

Harper et al. [13] found similar associations between condom non-use and first sexual intercourse before the age of 13, drinking alcohol, and using drugs, suggesting more pronounced declines among male than female students whose first sexual intercourse was before the age of 13. Similarly, our results revealed that for both sexes, condom non-use was highly associated with risk behaviors such as very early age of first sexual intercourse and substance use. Our study indicated that early age of sexual debut was associated with condom non-use. Brahmbhatt et al. [8] evaluated adolescents aged 15–19 years and verified that among both males and females, early age of sexual debut was a significant determinant of pregnancy. Magnusson et al. [36] developed a study in the US using data from the National Survey of Family Growth collected from 7356 women aged 15–44 years and concluded that early age of sexual debut is associated with inconsistent or non-use of contraceptives in later life.

Findings on age and the number of sexual partners are noteworthy in the context of condom non-use, as these differed between sexes: increasing age and number of sexual partners indicated less condom use for girls, while for boys, increasing age and number of sexual partners indicated higher condom use. Young, Burke, and Gabhainn [16] found that condom non-use among boys increased with age. Their results may be because young men reported authoritarianism and a need for power and control in the domain of condom-use and decision-making compared to their women partners due to gender and cultural norms [37]; it is possible that men are more coercive than women in terms of condom negotiation and that women may be giving in to men’s pressure [35]. This coercion may be intensified in cases of body dissatisfaction or others psychosocial problems [38]. Finally, coercion could be related to the absence of dialogue about sexual matters, and the fact that men almost always have the final word. Therefore, programs need to encourage condom use and training in negotiation for adolescents of both sexes. Education should also contain gender-specific messages, such as girls’ carrying or suggesting the use of condoms and learning ways to manage sexual pressure and authoritarian and abusive practices, and boys’ relinquishing the need for power and control.

Although starting long-term hormonal contraception was associated with a decrease in condom use among adult women [39], the use of other contraceptive methods, whether hormonal or not, was a protective factor against not using condoms in our study. This can be explained by the widespread understanding of the importance of dual protection [40]. In contrast to our findings, Goldstein et al. [41] conducted a prospective cohort study among girls aged 15–24 years and found that after starting a hormonal method, condom use decreased.

Being dissatisfied with one’s body, having an altered body perception, and reporting poor health are associated with lowered condom use self-efficacy, corroborating with data in the literature [38, 42, 43]. Although there is little literature on this subject, it seems that individuals who have body dissatisfaction or others psychosocial problems may be afraid of abandonment or rejection and therefore do not insist on using condoms. In addition, individuals with elevated body dissatisfaction may have increased anxiety and concern in the context of sexual intercourse. Consequently, they may lack assertiveness in broaching the topic of condoms and may be less likely to initiate conversations about safer sex practices [38].

These results indicate that education and preventive programs should take into account both health aspects and gender equity. This is apparent from our school and health service results, which also show that poor self-reported general health is strongly related to condom non-use. Physiological and psychosocial health perception are important to the student and can impact sexual behavior. According to Sarkar et al. [35], anxiety and depression were both negatively associated with condom use. Moreover, particularly for girls, condom use was predicted by higher quality of life, whereas taking medication for physical and psychological symptoms was associated with condom non-use [8, 44]. In this context, both school and health services have a relevant role in the sexual behavior of adolescents.

Our study had some limitations. Because it was cross-sectional, we cannot infer causality. The data were self-reported and may have been influenced by social desirability biases. Our study did not evaluate two important explanatory variables, “having concurrent partners” and “ever having had STIs,” which should be addressed in future research. Finally, in addition to evaluating the frequency of substance use, future studies should also examine the amount consumed in each occasion. Despite these limitations, our findings suggest that public health and clinical efforts to increase condom use among young people are warranted.

## Conclusion

High condom non-use is associated with no health care access and sexual health education, poor sexual practices, substance use, and poor self-perception, indicating areas for health promotion programs. Educating adolescents on the importance of condom use for STI prevention is critical to contraceptive counseling. Given the significant morbidity associated with STI acquisition, and to improve pregnancy prevention, health educators and clinicians should encourage condom use in adolescents. Programs should attempt to address behaviors strongly related to condom non-use, such as not seeking health services or professional health care and issues such as not receiving guidance on pregnancy prevention, AIDS, or other STIs. Public policies must be continuously updated and monitored to improve school and public health environments in order to promote healthy sexual behavior among adolescents.

## Data Availability

All data generated or analyzed during this study are included in this published article.

## Abbreviations

PeNSE: Brazilian National School-Based Health Survey
PR: Prevalence ratio
SD: Standard deviation

## References

[1] Idowu AA, Moyaki MG, Goon DT, Avramovic G, Lambert JOVA (2018). High rate of unplanned pregnancy in the context of integrated family planning and HIV care services in South Africa. BMC Health Serv Res.

[2] Raidoo S, Kaneshiro B (2015). Providing contraception to adolescents. Obstet Gynecol Clin N Am.

[3] Santelli BJ, Rochat R, Hatfield K, Gilbert C, Curtis K, Cabral R (1999). The Measurement and Meaning of Unintended Pregnancy.

[4] Ganchimeg T, Mori R, Ota E, Koyanagi A, Gilmour S, Shibuya K (2013). Maternal and perinatal outcomes among nulliparous adolescents in low- and middle-income countries: a multi-country study. BJOG An Int J Obstet Gynaecol.

[5] Gipson JD, Koenig MA, Hindin MJ, Gipson D, Michelle J (2008). The Effects of Unintended Pregnancy on Infant, Child, and Parental Health: A Review of the Literature. Stud Fam Plann.

[6] Moreno R, Nababan HY, Ota E, Wariki WM, Ezoe S, Gilmour S, et al. Structural and community-level interventions for increasing condom use to prevent the transmission of HIV and other sexually transmitted infections. Cochrane Database Syst Rev. 2014. 10.1002/14651858.CD003363.pub3. 10.1002/14651858.CD003363.pub3 PMC11184921 25072817

[7] Shafii T, Stovel K, Holmes K (2007). Association between condom use at sexual debut and subsequent sexual trajectories: a longitudinal study using biomarkers. Am J Public Health.

[8] Brahmbhatt H, Kågesten A, Emerson M, Decker MR, Olumide AO, Ojengbede O (2014). Prevalence and determinants of adolescent pregnancy in urban disadvantaged settings across five cities. J Adolesc Health.

[9] Casola AR, Nelson DB, Patterson F (2017). Sex differences in contraception non-use among urban Adolescents : risk factors. J Sch Health.

[10] Pinchoff J, Boyer CB, Mutombo N, Chowdhuri RN, Ngo TD (2017). Why don’t urban youth in Zambia use condoms? The influence of gender and marriage on non-use of male condoms among young adults. Price MA, editor. PLoS One.

[11] Woolley NO, Macinko J. Association between sociodemographic characteristics and sexual behaviors among a nationally representative sample of adolescent students in Brazil. Cad Saude Publica. 2019;35. 10.1590/0102-311x00208517. 10.1590/0102-311X00208517 30758456

[12] Green KM, Musci RJ, Matson PA, Johnson RM, Reboussin BA, Ialongo NS (2017). Developmental patterns of adolescent marijuana and alcohol use and their joint association with sexual risk behavior and outcomes in Young adulthood. J Urban Heal.

[13] Harper CR, Steiner RJ, Lowry R, Hufstetler S, Dittus PJ (2018). Variability in condom use trends by sexual risk behaviors. Sex Transm Dis.

[14] Eversole JS, Berglas NF, Deardorff J, Constantine NA (2017). Source of sex information and condom use intention among Latino adolescents. Heal Educ Behav.

[15] Schumacher A, Marzell M, Toepp AJ, Schweizer ML (2018). Association between marijuana use and condom use: a meta-analysis of between-subject event-based studies. J Stud Alcohol Drugs.

[16] Young H, Burke L, Gabhainn SN (2018). Sexual intercourse , age of initiation and contraception among adolescents in Ireland : findings from the Health Behaviour in School-aged Children ( HBSC ) Ireland study.

[17] Intituto Brasileiro de Geografia e Estatística (2017). Pesquisa Nacional de Saúde do Escolar - PeNSE.

[18] de Oliveira MM, Campos MO, de Andreazzi MAR, Malta DC, de Oliveira MM, Campos MO (2017). Características da Pesquisa Nacional de Saúde do Escolar - PeNSE. Epidemiol e Serviços Saúde.

[19] PRES N, Noll M, de Abreu LC, Baracat EC, Silveira EA, ICE S (2019). Ultra-processed food consumption by Brazilian adolescents in cafeterias and school meals. Sci Rep.

[20] Silva RMA, Andrade AC de S, Caiaffa WT, Medeiros DS de, Bezerra VM. (2020). National Adolescent School-based Health Survey - PeNSE 2015: sedentary behavior and its correlates. PLoS One.

[21] Dos Santos CC, Flores TR, Wendt A, Neves RG, MCF A, Santos IS (2018). Sedentary behavior and consumption of ultra-processed foods by Brazilian adolescents: Brazilian National School Health Survey (PeNSE), 2015. Cad Saude Publica.

[22] Noll M, ES NPR, Tiggemann CL, Custodio DC, Silveira EA (2020). Health-risk behavior differences between boarding and non-resident students: Brazilian adolescent National School Health Survey. Arch Public Heal.

[23] de Antunes H (2018). A, Rivadeneira-Guerrero MF, Goulart BNG de, Oenning NSX. Familiar factors and illicit drug use among Brazilian adolescents: an analysis of the Brazilian National Survey of school health (PeNSE, 2015). Cad Saude Publica..

[24] Escobar DFSS, ES NPR, de Jesus TF, Noll M (2020). Assessing the Mental Health of Brazilian Students Involved in Risky Behaviors. Int J Environ Res Public Health.

[25] de Oliveira MM, Campos MO, de Andreazzi MAR, Malta DC, de Oliveira MM, Campos MO (2017). Characteristics of the National Adolescent School-based Health Survey – PeNSE, Brazil. Epidemiol e Serviços Saúde.

[26] von Elm E, Altman DG, Egger M, Pocock SJ, Gøtzsche PC, Vandenbroucke JP (2007). Strengthening the reporting of observational studies in epidemiology (STROBE) statement: guidelines for reporting observational studies. BMJ..

[27] Barros AJ, Hirakata VN (2003). Alternatives for logistic regression in cross-sectional studies: an empirical comparison of models that directly estimate the prevalence ratio. BMC Med Res Methodol.

[28] Sun GW, Shook TL, Kay G (1996). Inappropriate use of bivariable analysis to screen risk factors for use in multivariable analysis. J Clin Epidemiol.

[29] Harrell FE (2001). Regression modeling strategies: with applications to linear models, logistic regression and survival analysis.

[30] Hodder RK, Homer S, Freund M, Bowman JA, Lecathelinais C, Colyvas K (2018). The association between adolescent condom use and individual and environmental resilience protective factors. Aust N Z J Public Health.

[31] Shrier LA, Harris SK, Sternberg M, Beardslee WR (2001). Associations of depression, self-esteem, and substance use with sexual risk among adolescents. Prev Med (Baltim).

[32] Thamotharan S, Grabowski K, Stefano E, Fields S (2015). An examination of sexual risk behaviors in adolescent substance users. Int J Sex Heal.

[33] Hansen BT, Kjær SK, Munk C, Tryggvadottir L, Sparén P, Hagerup-Jenssen M (2010). Early smoking initiation, sexual behavior and reproductive health - a large population-based study of Nordic women. Prev Med (Baltim).

[34] Johnson MW, Herrmann ES, Sweeney MM, LeComte RS, Johnson PS (2017). Cocaine administration dose-dependently increases sexual desire and decreases condom use likelihood: the role of delay and probability discounting in connecting cocaine with HIV. Psychopharmacology.

[35] Sarkar NN (2008). Barriers to condom use. Eur J Contracept Reprod Heal Care.

[36] Magnusson BM, Masho SW, Lapane KL (2012). Early age at first intercourse and subsequent gaps in contraceptive use. J Women’s Heal.

[37] Vasilenko SA, Kreager DA, Lefkowitz ES (2015). Gender, contraceptive attitudes, and condom use in adolescent romantic relationships: a dyadic approach. J Res Adolesc.

[38] Blashill AJ, Safren SA (2015). Body dissatisfaction and condom use self-efficacy: A meta-analysis. Body Image.

[39] Cushman LF, Romero D, Kalmuss D, Davidson AR, Heartwell S, Rulin M (1998). Condom use among women choosing long-term hormonal contraception. Fam Plan Perspect.

[40] Diniz SG, D’Oliveira AFPL, Lansky S (2012). Equity and women’s health services for contraception, abortion and childbirth in Brazil. Reprod Health Matters.

[41] Goldstein RL, DM U, Raine TR (2013). With Pills, Patches, Rings, and Shots: Who Still Uses Condoms? A Longitudinal Cohort Study. J Adolesc Health.

[42] Corona R, Hood KB, Haffejee F (2019). The relationship between body image perceptions and condom use outcomes in a sample of south African emerging adults. Prev Sci.

[43] Gillen MM, Lefkowitz ES, Shearer CL (2006). Does body image play a role in risky sexual behavior and attitudes?. J Youth Adolesc.

[44] Yakubu I, Salisu WJ. Determinants of adolescent pregnancy in sub-Saharan Africa: a systematic review. Reprod Health. 2018;15. 10.1186/s12978-018-0460-4. 10.1186/s12978-018-0460-4 PMC5787272 29374479

